# Relationship between toxoplasmosis in pregnant women and in their feline pets

**DOI:** 10.1186/1471-2334-13-S1-P25

**Published:** 2013-12-16

**Authors:** Andrei Csep, Radu Spineanu, Sonia Drăghici, Simona Cheregi

**Affiliations:** 1 University of Oradea, Romania

## Background

Toxoplasmosis is a parasitic disease caused by the protozoan *Toxoplasma gondii*. The infection is most commonly acquired through contact with cats and their feces or with raw or undercooked meat.

## Methods

Our goal was to study the relationship between pregnant women diagnosed with acute toxoplasmosis and the presence of this parasite in their house pets. Using chemiluminescence enzyme immunoassay (CLIA) we determined the presence of toxoplasma IgM and IgG antibodies and by enzyme immunoassay (EIA) toxoplasma IgA antibodies. Enzyme-linked immunosorbent assay (ELISA) was used to determine the presence of IgM and IgG toxoplasma antibodies in the cats belonging to the patients with confirmed acute toxoplasmosis.

## Results

The study included 240 pregnant women suspected with *Toxoplasma gondii* infection, during the years 2009-2012. Out of the pregnant women studied, 35 (14.5%) were diagnosed with acute toxoplasmosis, 61 (25.4%) of them had protective antibodies, while 144 (60%) had completely negative serology. More than half of the patients with acute toxoplasmosis (51.4%) respectively 18 patients, had owned cats. Of these, we found IgM to IgG toxoplasma seroconversion only in 6 (33.3%) cats, which were the direct infected ones, while 4 other cats (22.2%) had protective IgG antibodies, attesting an acute phase in the recent history. These could represent a source of infection for pregnant women by oocysts excreted in the external environment in their acute phase of the disease.

## Conclusion

Ten (28.5%) of the 35 pregnant women diagnosed with acute toxoplasmosis could have been contaminated by the cats they owned, which were found to be in the acute phase of the disease, or by previous oocysts excreted in the external environment by the cats with protective antibodies in the moment of their examination.

